# Monodisperse and Nanometric-Sized Calcium Carbonate Particles Synthesis Optimization

**DOI:** 10.3390/nano12091494

**Published:** 2022-04-28

**Authors:** Francesca Persano, Concetta Nobile, Clara Piccirillo, Giuseppe Gigli, Stefano Leporatti

**Affiliations:** 1 Department of Mathematics and Physics, University of Salento, 73100 Lecce, Italy; giuseppe.gigli@unisalento.it; 2 CNR Nanotec—Institute of Nanotechnology, 73100 Lecce, Italy; concetta.nobile@nanotec.cnr.it (C.N.); clara.piccirillo@nanotec.cnr.it (C.P.)

**Keywords:** calcium carbonate nanoparticles, vaterite, nanomedicine, drug delivery, resveratrol

## Abstract

Calcium carbonate (CaCO_3_) particles represent an appealing choice as a drug delivery system due to their biocompatibility, biodegradability, simplicity and cost-effectiveness of manufacturing, and stimulus-responsiveness. Despite this, the synthesis of CaCO_3_ particles with controlled size in the nanometer range via a scalable manufacturing method remains a major challenge. Here, by using a co-precipitation technique, we investigated the impact on the particle size of different synthesis parameters, such as the salt concentration, reaction time, stirring speed, and temperature. Among them, the salt concentration and temperature resulted in having a remarkable effect on the particle size, enabling the preparation of well-dispersed spherical nanoparticles with a size below 200 nm. Upon identification of optimized synthesis conditions, the encapsulation of the antitumoral agent resveratrol into CaCO_3_ nanoparticles, without significantly impacting the overall size and morphology, has been successfully achieved.

## 1. Introduction

Drug delivery systems have been widely explored in a range of biomedical applications and, particularly, for cancer therapy. These anti-cancer drugs tend frequently to be insoluble in water or biological media, and do not possess a target-specific effect, thus exerting their cytotoxic effect not only on tumor cells but also on healthy cells, leading to several collateral effects [1,2,3]. To overcome these issues, research over the past decades has focused on the development of new delivery systems that can improve the solubility, biodistribution, and tumor targeting-ability of anti-cancer drugs [4,5]. Nanotechnology has the potential to enhance the therapeutic efficacy of conventional anticancer drugs by increasing their stability and solubility and enabling the precise delivery of therapeutics within a specific tissue or organ [6]. Although a wide number of nanomaterials have been explored in preclinical models, biodegradable materials are often preferred over non-biodegradable ones for biomedical applications since they generally exhibit an improved toxicity profile [7,8]. Among these biodegradable nanomaterials, calcium carbonate (CaCO_3_) is particularly attractive for the development of nanocarriers due to its unique properties, including high biocompatibility and pH-responsiveness, along with the simplicity and low cost of production [9,10]. The pH-responsiveness of CaCO_3_ nanoparticles (CaCO_3_NPs) makes them particularly appealing for cancer treatment since acidosis is a hallmark of the tumor microenvironment [11]. Importantly, to fully exploit the potential of CaCO_3_-based platforms for therapeutic applications, the particle size must be kept in the nanoscale range. Particle size is indeed one of the major parameters affecting biodistribution and consequently determining the therapeutic outcome of nano-formulated drugs [12,13,14]. This is valid even if the particles are locally administrated (i.e., intratumorally), since particle size may alter their retention rate, local distribution, and penetration capability [15].

Different approaches have been investigated for preparing spherical CaCO_3_NPs with a controlled size in the nanoscale range. A common strategy is to include polymeric additives, such as poly (acrylic) acid (PAA) and polydopamine (PDA) in the reaction mixture, thus limiting crystal growth [16,17].

The chemical precipitation of salt precursors, such as CaCl_2_ and Na_2_CO_3_, represents the most utilized approach for the synthesis of CaCO_3_-based materials [18,19]. The immediate nucleation of CaCO_3_ particles is achieved by the vigorous mixing of the saline solutions of CaCl_2_ and Na_2_CO_3_. From the aqueous solution, CaCO_3_ precipitates three anhydrous crystalline polymorphs (rhomboidal calcite, needle aragonite, and spherical vaterite), two hydrated forms (monohydrate hexahydrate ikaite), and an amorphous phase. Calcite is the stable polymorph, while vaterite and aragonite are the metastable forms that easily transform into the stable form. Among the anhydrous polymorphs, spherical particles of vaterite are employed in a variety of applications due to their increased water solubility and unique chemical, physical, and mechanical properties, and reduced chemical and biological inertness [20]. Synthesis parameters, including reactant concentrations, temperature, and the type of solvent can influence the abundance of each morphological form of CaCO_3_. Generally, high levels of supersaturation and moderate temperatures (25–45 °C) favor the formation of polymorph vaterite [19].

Despite the number of studies conducted so far, the control of size and morphology using a scalable synthesis method remains a challenge for the development of CaCO_3_-based nanocarriers [21,22].

In this study, we have exhaustively investigated the impact of the different synthesis parameters, such as the mixing speed, precursor concentration, and temperature on the size and morphology of the synthesized CaCO_3_ particles. Interestingly, using only the tuning synthesis parameters, we were able to form particles with a size confined to the nanometer range. Optimized synthesis conditions were finally applied to test drug encapsulation in CaCO_3_NPs using resveratrol as a drug model.

## 2. Materials and Methods

### 2.1. Materials

Calcium chloride dihydrate (CaCl_2_ × 2H_2_O), ≥99%; Sodium carbonate (Na_2_CO_3_), BioXtra, ≥99.0%; Ethylene glycol (EG), Reagent Plus, ≥99%; Resveratrol (C_14_H_12_O_3_), ≥99% (HPLC); Acetone; Ethanol; and Methanol were purchased from Sigma-Aldrich (St. Louis, MO, USA).

### 2.2. Cell Lines

A human U87 GBM cell line was obtained from ATCC. Cells were maintained in Dulbecco’s Modified Eagle’s Medium (DMEM) supplemented with 10% fetal bovine serum (FBS), 50 U/mL penicillin, and 250 μg/mL streptomycin (Sigma-Aldrich, St. Louis, MO, USA). All cells were maintained in an incubator at 37 °C with 5% CO_2_.

### 2.3. In Water/EG, 1:5 (v/v): Stirring Speed

The synthesis of CaCO_3_ NPs was carried out following the amended method reported by Thapa et al. (2017) [23]. In particular, a double decomposition reaction was carried out, mixing equal volumes of a 0.1 M solution of CaCl_2_·2H_2_O and Na_2_CO_3_, with each of them prepared in water and ethylene glycol (1:5, *v*/*v*) and left under magnetic stirring for 30 min. Subsequently, the synthesized NPs were collected via sequential washing with ethanol, methanol, and acetone at 10,000 rpm for 10 min to remove unreacted ions and cosolvent molecules, and then dried at 60 °C for 1 h. The synthesis was repeated using different stirring speeds, such as 625, 750, 825, 1000, and 1125 rpm.

### 2.4. In Water/EG, 1:5 (v/v): Reaction Time

Since vaterite is a metastable polymorph of CaCO_3_, when an aqueous saline solution free of additives is the synthesis means of the CaCO_3_ crystals, by prolonging the incubation time, there is often a reduction in the content of vaterite [19,24]. It has been reported that, normally, the time required for the transformation of metastable vaterite into calcite is between a few minutes and several hours [25,26]. To evaluate the impact of the reaction time on the size and morphology of the CaCO_3_ crystals synthesized in water and EG (1:5, *v*/*v*), the synthesis was carried out using a magnetic stirring speed of 1125 rpm, varying the reaction time: 30, 60, 120, and 180 min. The other experimental conditions were kept unchanged.

### 2.5. In Water/EG, 1:1, 1:3 and 1:5 (v/v)

The polyols added to the reaction mixture can stabilize the vaterite nuclei by limiting their subsequent transformations, thanks to the increase in supersaturation and the formation of a three-dimensional network of hydrogen-bonded molecules [27]. Here the impact of the amount of polyol added on the size and morphology of the CaCO_3_ crystals was studied. Vaterite was synthesized in a series of experiments by adding different amounts of EG to the solutions of CaCl_2_·2H_2_O and Na_2_CO_3_ and then subjecting them to vigorous magnetic stirring at 1125 rpm for 30 min. Co-precipitation experiments were conducted at room temperature using the prepared 0.1 M saline solutions in water and EG, 1:1, 1:3, and 1:5 (*v*/*v*), respectively.

### 2.6. In Water/EG, 1:5 (v/v): Initial Concentration of Precursors

It has been reported that for CaCO_3_ particles synthesized in pure water, the diameter significantly depends on the salt concentration. In particular, a higher concentration of salts leads to an increase in supersaturation, which translates into an increase in the nucleation speed resulting in smaller crystal sizes at the same total precipitate mass [28,29]. Here, the effect of the initial precursor concentration on the size of the synthesized particles in a mixture of water and EG was evaluated. For our study, we used solutions of CaCl_2_·2H_2_O and Na_2_CO_3_ prepared in water and EG (1:5, *v*/*v*) at a concentration of 0.1, 0.05, 0.025, and 0.01 M, respectively. Equal volumes of the two solutions were mixed and left under magnetic stirring at 1125 rpm for 30 min.

### 2.7. In Water/EG, 1:5 (v/v): At 4 °C

We studied the effect of temperature on the morphology and size of CaCO_3_ particles, synthesized in a mix of water and ethylene glycol (1/5, *v*/*v*), in a cold room at a temperature of 4 °C. The CaCl_2_·2H_2_O and Na_2_CO_3_ salts were dissolved in water/EG at concentrations of 0.01, 0.025, and 0.05 M. Equal volumes of the two solutions were mixed under vigorous magnetic stirring at 1125 rpm for 20 and 24 h.

### 2.8. Encapsulation Efficiency (EE) of Resveratrol into CaCO_3_NPs

The large surface of the vaterite crystals allows the absorption of a high quantity of active molecules [30]. Here the encapsulation efficiency was evaluated using resveratrol (Res) as a model drug. For the loading of the Res into the vaterite particles, the active molecules were added to the reaction mixture. The reaction was carried out by mixing equal volumes of 40 mM CaCl_2_·2H_2_O and 40 mM resveratrol (Res) solutions followed by incubation for 10 min at room temperature under magnetic stirring at 1125 rpm. Subsequently, a double volume of 20 mM Na_2_CO_3_ solution was added to the reaction vial and the mixture was left under magnetic stirring for a further 30 min at 1125 rpm. All solutions were prepared in water/EG (1:5, *v*/*v*). After centrifugation, the supernatant was recovered, and the encapsulation efficiency (EE) was determined spectrophotometrically (λ = 303 nm) by an indirect method using the following formula: Encapsulation efficiency (EE)% = (total amount of drug added—amount of drug in supernatant)/total amount of drug added × 100. Similarly, the EE of the Res was determined by conducting the synthesis at 4 °C.

### 2.9. Morphological Characterization

CaCO_3_ particle size and morphology were analyzed by the transmission electron microscopy (TEM) technique. CaCO_3_ samples were imaged by a JEOL JEM 1011 TEM microscope (JEOL USA, Inc., Peabody, MA, USA), operated at an acceleration voltage of 100 kV. CaCO_3_ samples were prepared by depositing a droplet of ethanol-dispersed particles onto a standard C-coated Cu grid. The average size of particle samples, as imaged by TEM, was determined by the ImageJ software (*ImageJ*, version 1.52t, Free Software for Image Data Analysis, imagej.nih.gov/ij/index.html, accessed on 22 March 2022). The number of analyzed particles exceeded 250 in each sample. Each point plotted on the graphs represents the average of 3 samples obtained under the same conditions.

### 2.10. Phase Composition

The phase composition of selected samples was determined through X-ray Diffraction (XRD) and FTIR. XRD patterns were acquired with an X’ Pert PRO MRD diffractometer, (Malvern Panalytical Ltd., Malvern, UK) equipped with a fast RTMS detector, using a CuK α radiation (40 kV and 40 mA). Data were recorded in the 20–60° 2θ range, with a virtual step-scan of 0.005° 2θ, and a counting time of 100 s. Phase identification was performed compared to the standard JCPDF diffraction patterns 00-005-0586 for CaCO_3_ calcite and 00-033-0268 for CaCO_3_ vaterite. The relative crystalline phase composition was estimated by comparing the areas of the 100% peaks (after background subtraction); the considered peaks were located at 29.4° for calcite and 32.5° for vaterite.

FTIR spectra were measured with FT/IR-6000 Jasco (Jasco Europe, Cremella, Italy) in transmission mode; the spectra were acquired on a disc made of approximately 2 mg of powder and 200 mg of KBr.

### 2.11. In Vitro Release Study

The in vitro degradation of the CaCO_3_ particles at different pH values was tested in buffers of pH 7.4, 6.5, and 5.5 at 37 °C. The CaCO_3_ crystals (5 mg) were resuspended in 2 mL of the buffer solution. At different time points, the release of Res was determined by measuring the absorbance in the collected supernatants at the wavelength of 303 nm.

### 2.12. MTT Assay (Cell Viability)

Cells were grown in 96-well plates in 200 μL of medium volume per well and incubated with different concentrations of CaCO_3_NPs. After treatment, the medium was removed, and cells were washed twice with PBS (pH 7.4) at 37 °C and placed into PBS. Then, the MTT solution was added to the cells at a concentration of 1 mg/mL, and the cells were incubated for 3 h under standard conditions. After incubation, PBS was removed and DMSO was added to dissolve formed formazan crystals. The optical density of formazan solution in DMSO was measured at 540 nm.

## 3. Results and Discussion

### 3.1. Impacts of Mixing Speed on CaCO_3_NPs Size

The co-precipitation of calcium and carbonate salts in an aqueous solution is a simple and convenient method for the synthesis of CaCO_3_NPs. However, this method usually results in the formation of particles in the micrometer range in the absence of stabilizer agents [31]. The stirring speed at which the two saline solutions are mixed is a key factor in the precipitation process. In the aqueous medium, the activation energy of the nucleation of the vaterite particles is influenced by the intensity of the agitation. The local inhomogeneity of the supersaturation nucleation can have an impact on the formation of favorable conditions for the precipitation of pure vaterite crystals [32].

**Figure 1:**
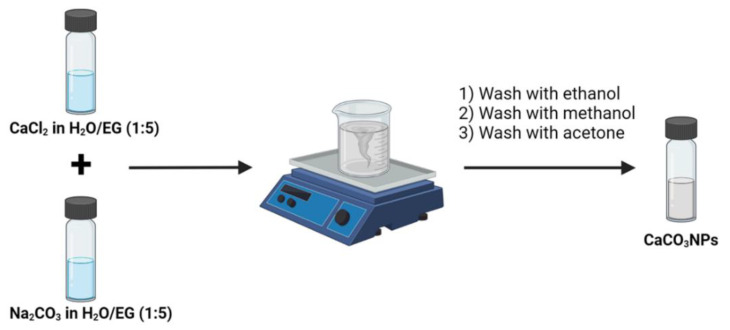
Schematic illustration of the procedure used for the fabrication of CaCO_3_NPs.

We investigated the impact of mixing speed on the physicochemical characteristics, such as the size and morphology, of CaCO_3_NPs obtained following a coprecipitation technique. The procedure utilized for CaCO_3_NPs manufacturing is described in Figure 1.

**Figure 2:**
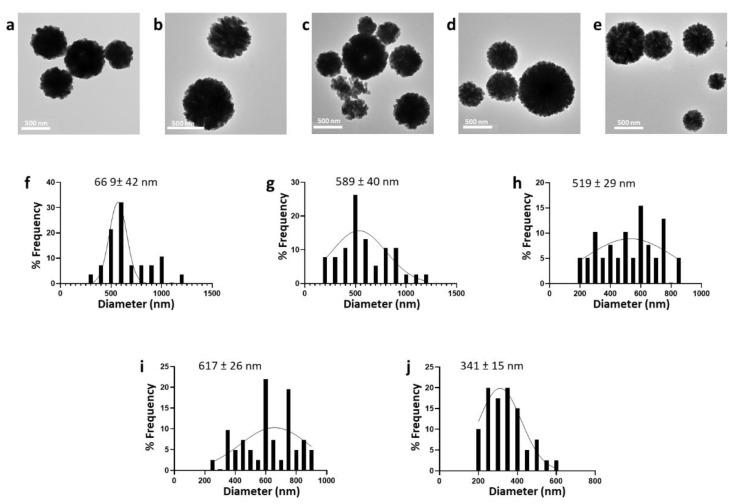
TEM images (**a**–**e**) and relative size distribution (**f**–**j**) of CaCO_3_NPs prepared by coprecipitation using different stirring rates, such as 625 rpm (**a**,**f**), 750 rpm (**b**,**g**), 875 rpm (**c**,**h**), 1000 rpm (**d**,**i**), 1125 rpm (**e**,**j**).

In this scope, CaCO_3_NPs were obtained following a coprecipitation technique using different stirring rates (625, 750, 875, 100, 1125 rpm) as shown in the TEM images of Figure 2a–e. The particle size distribution and average, as determined by ImageJ software, are shown in Figure 2f–j; here, size is reported as the mean ± SEM. Such data showed an inverse correlation between the nanoparticle size and mixing speed, with an average size that decreased from 669 ± 42 nm to 341 ± 15 nm by simply increasing the stirring speed. Interestingly, we noticed that size reduction was accompanied by an improvement in the size distribution (Figure 2).

### 3.2. Effect of Reaction Time on CaCO_3_NP Size and Phase Composition

Vaterite is a metastable polymorph of CaCO_3_, therefore the prolongation of the reaction time leads to a reduction of the vaterite content when the synthesis medium is an aqueous solution free of organic additives [33]. The remaining precipitated vaterite crystals in aqueous solutions recrystallize into the more stable polymorphs of CaCO_3_, namely aragonite and calcite. Normally, the time required for the transformation of vaterite into calcite is between a few minutes and several hours [34].

**Figure 3:**
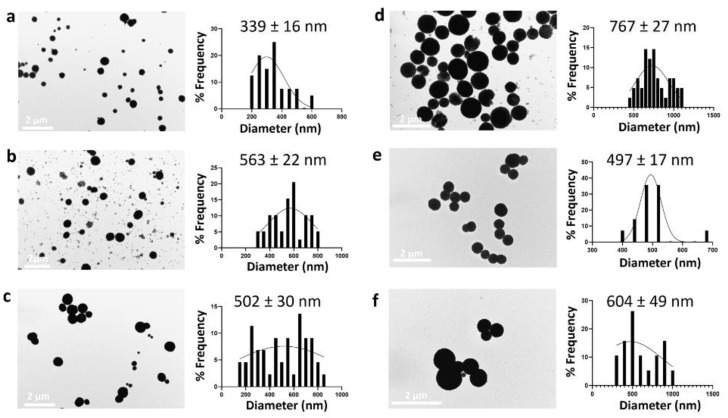
TEM images (left) and size distribution (right) of CaCO_3_NPs prepared using different reaction times, such as 30 min (**a**), 1 h (**b**), 2 h (**c**), and 3 h (**d**), and reducing the H_2_O/EG ratio at 1:3 (**e**) and 1:1 (**f**).

In our study, as previously reported, we showed that the presence of organic solvents such as ethylene glycol (EG) can prevent the complete transformation of vaterite into calcite, thus maintaining a spherical morphology (Figure 3) [34]. Prolongation of the reaction time from 30 min to 3 h led to an increase in the particle size from 339 ± 16 nm to 767 ± 27 nm. A reaction time of less than 30 min was not sufficient for the nucleation and growth of CaCO_3_NPs (data not shown).

We also confirmed the importance of EG for controlling particle size by reducing the ratio of H_2_O:EG to 1:3 and 1:1. The decrease in the amount of EG in the reaction medium resulted in the formation of nanoparticles with an average size of 497 ± 17 nm and 604 ± 49 nm using an H_2_O:EG ratio of 1:3 and 1:1, respectively (Figure 3e,f).

**Figure 4:**
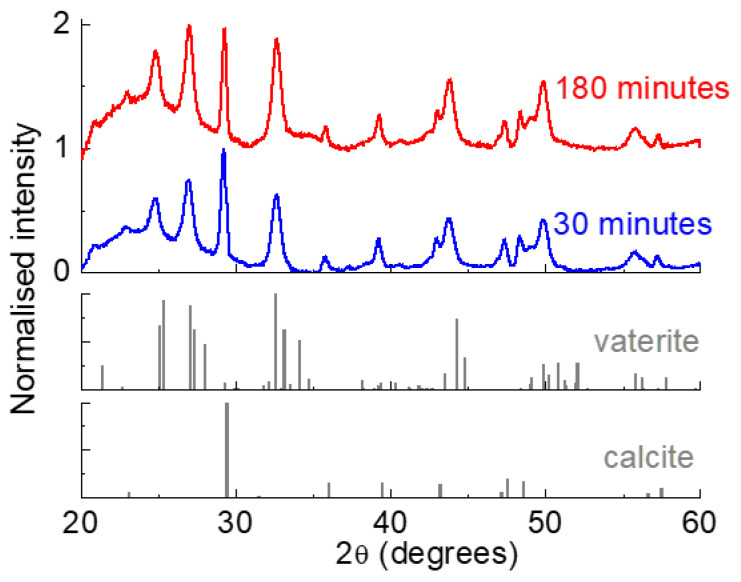
XRD patterns for samples prepared in W:EG 1:5 for 30 and 180 min. The acquired data are compared to the standard patterns for calcite and vaterite.

To determine the effect of different reaction times on the phase composition, XRD patterns were acquired for CaCO_3_NP prepared for 30 and 180 min; the normalized diffraction patterns for both samples are shown in Figure 4.

In both cases, two polymorphs of calcium carbonate can be detected, namely vaterite and calcite. The relative proportion of each phase, however, is different according to the reaction time (see Table 1). In the sample prepared for 30 min, in fact, vaterite is approximately 58%wt; increasing the time to 3 h leads to a significant increase in this phase to 67%wt.

These results may seem surprising, since vaterite is a metastable phase, which tends to convert to more stable phases, such as calcite with more prolonged reaction times [19,34]. It must be highlighted, however, that such conversion can be affected by different parameters, including the presence of organic molecules and/or other ions [35]. Moreover, other studies showed that longer reaction times may lead to a higher vaterite content [26]. Indeed, the formation of different phases between the different CaCO_3_ polymorphs is a rather complex topic, with many parallel processes occurring and different elements to be considered.

**Figure 5:**
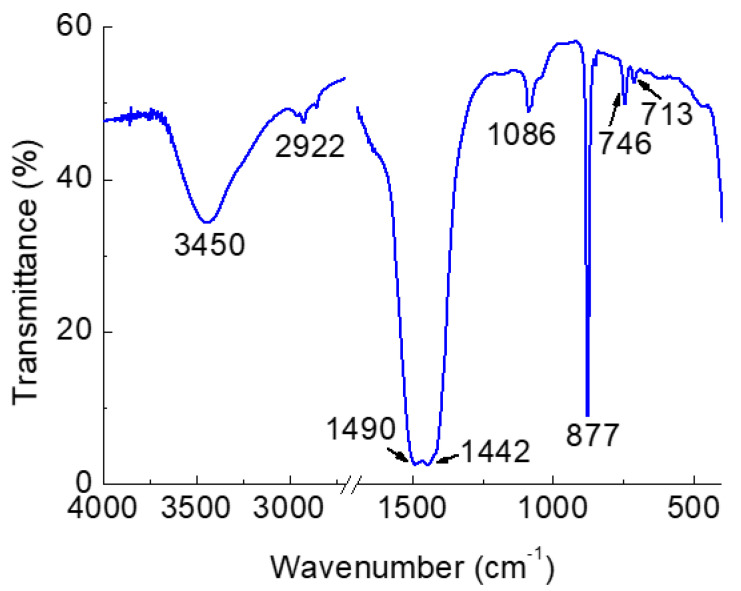
FTIR spectrum of the sample W:EG 1:5, 30 min.

To assess whether any significant organic contamination was present in the CaCO_3_NP, FTIR spectra were taken; Figure 5 shows, as an example, the spectrum for the sample prepared in W:EG 1:5 for 30 min. It can be seen that peaks characteristic of CaCO_3_ vaterite are present; indeed, signals at 746, 877, and 1086 cm^−1^ were detected. They correspond, respectively, to the υ4, υ3, and υ1 modes of vibration of the CO_3_^2−^ ion in the vaterite [36]. A smaller signal at 713 cm^−1^ was also registered; this can be attributed to the CO_3_^2−^ υ4 mode for calcite [37], indicating the presence of this phase too. The peaks at 1442 and 1490 cm^−1^, on the other hand, belong to the υ3 asymmetric mode, while the broad one at 3450 cm^−1^ corresponds to the OH stretching of water and/or residual solvents [36]. Indeed, the presence of organic residues can also be confirmed by the peak at 2922 cm^−1^, which belongs to the C-H vibration [38]. However, the intensity of the detected signal is very weak, indicating that the concentration of the residual organic solvent is very low, and that the majority of the solvent is removed through washing. Although FTIR is not a quantitative technique, it can be assumed that such an estimated low amount should not pose a threat of toxicity in the drug delivery processes.

### 3.3. Effect of the Initial Concentration of Precursors

**Figure 6:**
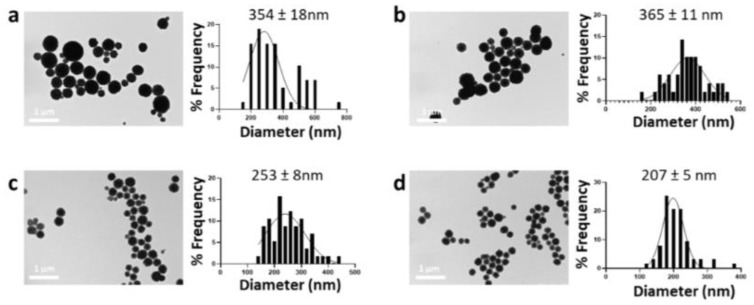
TEM images and size distribution of CaCO_3_NPs prepared with varying salt concentrations to 0.1 (**a**), 0.05 (**b**), 0.025 (**c**), and 0.01 M (**d**).

EG molecules have polar alcohol groups characterized by high cohesive energy toward the cationic Ca^2+^ ions in the solution. The strong association between Ca^2+^ ions and alcoholic groups determines a local increase in supersaturation so that nucleation occurs at a greater speed in the vicinity of these bound ions than the mass. The particle size of vaterite obtained in water/EG (1:5) was examined concerning the initial salt concentration. Our study revealed a positive correlation between the salt concentration (CaCl_2_ and Na_2_CO_3_) and the size of the synthesized nanoparticles (Figure 6). This effect may be related to a limited number of nucleation sites made available by polyol functional groups. When the crystalline nuclei occupy all the sites, it is energetically favorable for the remaining ions to associate with the growing particles rather than forming new nuclei. As a result, more ions are present in the solution and more ions will participate in the growth of CaCO_3_NPs.

Testing different salt concentrations, we identified 0.01 M as the optimal condition for ensuring the formation of nanoparticles with the smallest average size (207 ± 5 nm) and good size distribution.

### 3.4. Effect of Temperature on the Size of CaCO_3_NPs

In wet chemical nanoparticle synthesis, it is generally accepted that reaction temperature is one of the parameters that most impact a nanoparticle’s size, usually showing an inverse correlation between temperature and size.

**Figure 7:**
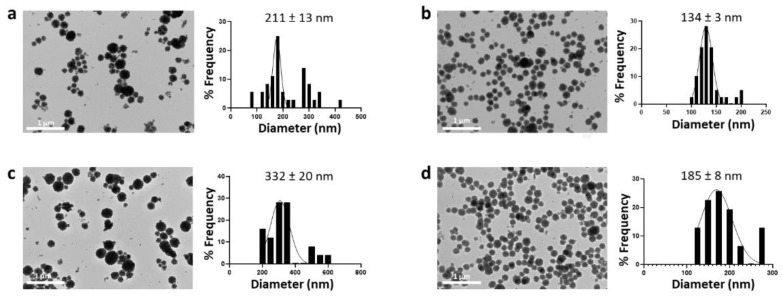
TEM images and size distribution of CaCO_3_NPs prepared at 4 °C varying the salt concentration (0.05 (**a**,**c**) and 0.025 M (**b**,**d**)) and the reaction time (20 h (**a**,**b**) and 24 h (**c**,**d**)).

CaCO_3_ is a “reverse soluble” compound that at higher temperatures is less soluble due to the poor solubility of carbon dioxide in water. It is also known that as the temperature increases, the nucleation rate increases [39]. All this suggests an advantage of the elevated temperatures in the precipitation of smaller vaterite crystals and the consequent prevention of the recrystallization process in more stable polymorphs. On the contrary, using salt concentrations of 0.050 and 0.025 M, by decreasing the synthesis temperature from room temperature to 4 °C, the average nanoparticle size decreased to 211 ± 13 nm and 134 ± 3 nm, respectively, with a simultaneous narrowing of the particle size distribution (Figure 7). However, the extension of the reaction time from previously optimized 30 min to 20 h and a minimal salt concentration of 0.025 M were necessary to ensure the formation of nanoparticles. Instead, a reaction time longer than 20 h resulted in an increase in the average CaCO_3_NP size.

Overall, our results suggest that a reaction temperature of 4 °C, a reaction time of 20 h, and a precursor concentration of 0.025 M were optimal to guarantee the formation of nanosized spherical vaterite structures.

### 3.5. Loading Efficiency and In Vitro Release

The most common technique for the encapsulation of drugs in CaCO_3_NPs consists of its coprecipitation during nanoparticle synthesis. Likely, drug incorporation inside CaCO_3_NPs relies on the establishment of an ionic interaction with Ca^2+^ ions and/or CO_3_^2−^ ions. Therefore, in this approach, the most relevant factors are steric effects, the molecular weight of the drug that is intended to be loaded, and its affinity for the Ca^2+^ and CO_3_^2−^ ions [40]. It is also possible to improve the loading efficiency by adjusting the pH of the reaction in a way to control the electrostatic or hydrophobic interaction between the drug and the ions that compose the nanoparticles [41].

**Figure 8:**
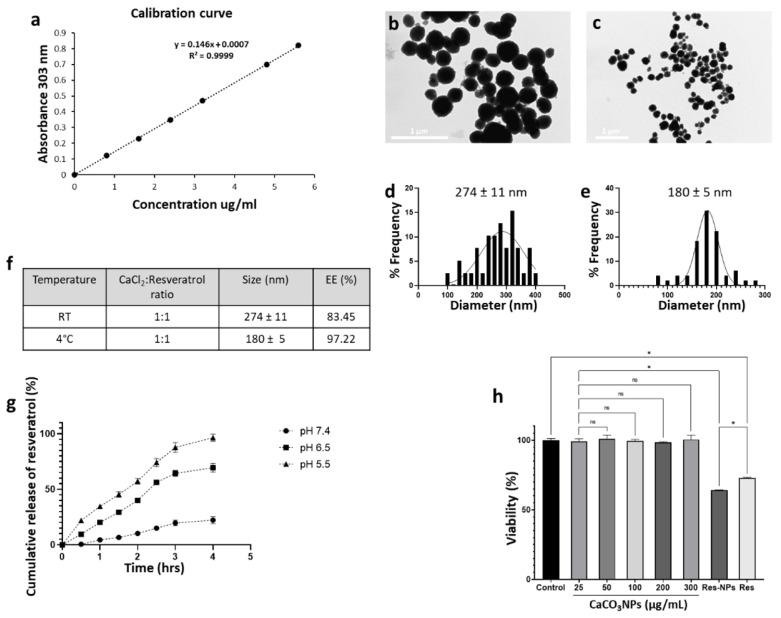
Preparation and characterization of Res-loaded CaCO_3_ NPs. Calibration curve of Resveratrol (**a**). TEM image and relative size distribution of Res-loaded CaCO_3_NPs synthesized at room temperature (**b**,**d**) and 4 °C (**c**,**e**). Average size and EE (%) for CaCO_3_NPs synthesized at RT and 4 °C (**f**). In vitro evaluation of Res release at different pH values (pH = 7.4, pH = 6.5, pH = 5.5) (**g**). MTT assay of U87 cells after 24 h of exposure to increasing concentrations of CaCO_3_NPs (25; 50; 100; 200; 300 ug/mL), 100 μM of Res loaded into CaCO_3_NPs (Res-NPs) and free (Res) (**h**). * *p* < 0.05; ns, not significant; one-way ANOVA test.

The loading efficiency was determined using resveratrol (Res) as a drug model. The entrapment of the drug into CaCO_3_NPs was confirmed by spectrophotometrically quantifying the amount of free drug remaining in the supernatant after washing (Figure 8). Using the optimized synthesis conditions determined for room temperature (salt concentration 0.01 M, reaction time 30 min, and mixing speed of 1125 rpm) and 4 °C (salt concentration 0.025 M, reaction time 24 h, and mixing speed of 1125 rpm) preparations, we were able to achieve a loading efficiency of 83.45% and 97.22%, respectively (Figure 8).

The biodegradability of nanomaterials is an essential prerequisite for their suitability in biomedical applications, ensuring their rapid elimination once they have completed their function. The peculiarity of CaCO_3_-based nanomaterials is that their degradation rate is accelerated at acidic pH, thereby allowing the release of the encapsulated drug in response to pH acidification [9].

The drug release profile of Res by CaCO_3_NPs in response to pH changes was assessed in phosphate buffer solutions of pH 7.4, 6.5, and 5.5. As shown in Figure 8g, around 96% of Res was released within 4 h in an acidic environment (pH 5.5), in a slightly acidic environment (pH 6.5) around 69% of Res was released, while at neutral pH (pH 7.4) only 22% RES was released within the same time. The results showed a pH-dependent release of the drug due to the accelerated degradation of CaCO_3_NPs in acidic environments (Figure 8g).

### 3.6. Evaluation of Toxicity

The cytotoxicity of the obtained CaCO_3_NPs with or without Res was assessed using a standard MTT assay, which estimates the percentage of alive cells after incubation with the different treatments. The effectiveness of Res-loaded CaCO_3_NPs in inhibiting cell proliferation was assessed in a human glioblastoma cell line (U87 cells) and a comparison was made with free Res and CaCO_3_NPs without the drug. In line with previous studies, no signs of toxicity were observed after the exposure to different concentrations of CaCO_3_NPs (from 25 to 300 μg/mL), confirming the high biocompatibility of CaCO_3_-based nanomaterials (Figure 8h). On the other hand, a significant reduction in cell viability was observed in U87 cells subjected to Res-loaded CaCO_3_NPs for 24 h. Indeed, after the 24-h incubation, the viability of U87 cells dropped to ~65%. Importantly, the cytotoxic effect induced by the treatment with CaCO_3_NPs containing Res was even more pronounced than those observed in U87 cells treated with the unformulated drug (~72%), corroborating the ability of nanocarriers to boost the therapeutic effect of conventional anticancer treatments (Figure 8h).

## 4. Conclusions

We presented a systematic optimization and evaluation of different synthesis parameters with the aim to identify the conditions that can allow the production of vaterite CaCO_3_ particles with an average size in the nanoscale range (≤200 nm).

Using an easily scalable synthesis setting, we identified reaction time, precursor concentrations, and temperature as the most important factors for controlling nanoparticle growth during the synthesis.

Optimized conditions allowed the preparation of CaCO_3_-based monodisperse spherical nanostructures with a size smaller than 200 nm. The synthesized nanoparticles were characterized by TEM. Due to their monodispersion and small size, the obtained nanoparticles are highly suitable for biomedical applications, including intravenous administration.

Efficient encapsulation of an anticancer drug (Res) was obtained in CaCO_3_NPs following a coprecipitation method. The drug release profile of Res-loaded CaCO_3_NPs showed that the release of Res is enhanced in acidic pH demonstrating that the developed system has the potential to enable controlled release of the payload. The pH-responsiveness of Res-loaded CaCO_3_NPs may help to prevent the undesired off-target release of the drug in organs such as the liver, heart, and spleen.

In the concentration range of 25–300 µg/mL, CaCO_3_NPs did not exhibit any toxicity in U87 glioblastoma cells. The inhibitory effect on cancer cell proliferation on U87 glioblastoma cells by Res-loaded CaCO_3_NPs turned out to be more potent than that of free Res at the concentration of 100 µM.

Our results indicate that we were able to synthesize CaCO_3_NPs with an average size suitable for biomedical applications, which holds great promise for further in vivo preclinical studies.

## Figures and Tables

**Table 1:** Relative phase composition for samples prepared in W:EG 1:5 for different times. All values are expressed in wt% and have an error of about 3%.

Reaction Time	30 Minutes	180 Minutes
Calcite (%)	41.5	32.5
Vaterite (%)	58.5	67.5

## Data Availability

The data presented in this study are available on request from the corresponding author.
